# Effectiveness of robot-assisted therapy on ankle rehabilitation – a systematic review

**DOI:** 10.1186/1743-0003-10-30

**Published:** 2013-03-21

**Authors:** Mingming Zhang, T Claire Davies, Shane Xie

**Affiliations:** 1 Department of Mechanical Engineering, University of Auckland, Auckland, New Zealand; 2 Department of Surgery, University of Auckland, Auckland, New Zealand

**Keywords:** Robot-assisted therapy, Ankle rehabilitation, Clinical effectiveness

## Abstract

**Objective:**

The aim of this study was to provide a systematic review of studies that investigated the effectiveness of robot-assisted therapy on ankle motor and function recovery from musculoskeletal or neurologic ankle injuries.

**Methods:**

Thirteen electronic databases of articles published from January, 1980 to June, 2012 were searched using keywords ‘ankle*’, ‘robot*’, ‘rehabilitat*’ or ‘treat*’ and a free search in Google Scholar based on effects of ankle rehabilitation robots was also conducted. References listed in relevant publications were further screened. Eventually, twenty-nine articles were selected for review and they focused on effects of robot-assisted ankle rehabilitation.

**Results:**

Twenty-nine studies met the inclusion criteria and a total of 164 patients and 24 healthy subjects participated in these trials. Ankle performance and gait function were the main outcome measures used to assess the therapeutic effects of robot-assisted ankle rehabilitation. The protocols and therapy treatments were varied, which made comparison among different studies difficult or impossible. Few comparative trials were conducted among different devices or control strategies. Moreover, the majority of study designs met levels of evidence that were no higher than American Academy for Cerebral Palsy (CP) and Developmental Medicine (AACPDM) level IV. Only one study used a Randomized Control Trial (RCT) approach with the evidence level being II.

**Conclusion:**

All the selected studies showed improvements in terms of ankle performance or gait function after a period of robot-assisted ankle rehabilitation training. The most effective robot-assisted intervention cannot be determined due to the lack of universal evaluation criteria for various devices and control strategies. Future research into the effects of robot-assisted ankle rehabilitation should be carried out based on universal evaluation criteria, which could determine the most effective method of intervention. It is also essential to conduct trials to analyse the differences among different devices or control strategies.

## Introduction

Tejima, 2000 [1] The human ankle joint is a very complex bony structure in the human skeleton and plays a significant role in maintaining body balance during ambulation [1]. In fact, the ankle is the most common site of sprain injuries in the human body, with over 23,000 cases estimated to occur per day in the United States [2] and about 100, 000 emergency department presentations per year in Australia [3]. In New Zealand, more than 82,000 new claims and 17,200 ongoing claims related to ankle injuries were made to the Accident Compensation Corporation (ACC) in the 2000/01 year, costing an estimated 31.8 million New Zealand dollars and making ankle related claims the fourth biggest cost to ACC [4]. Additionally, neurologic injuries like stroke, traumatic brain and spinal cord injuries are also leading causes for ankle disabilities. In the United States, at least 750, 000 incident and recurrent strokes occurred with the prevalence rate being about 200 to 300 patients per 100,000 inhabitants in 1995 [5]. However, the biggest effect on patients with ankle disabilities and their family members is usually a result of long-term impairment, limitation of activities and reduced participation.

Traditionally ankle injuries are rehabilitated via physiotherapy and however evidence suggests that without sufficient rehabilitation: 44% of people will have future problems [6,7]; ambulation is markedly compromised; re-injury prevalence is high; and approximately 38% of people will have recurrent activity limitations affecting their function [8]. Furthermore, during a rehabilitation treatment, cooperative and intensive efforts of therapists and patients are required over prolonged sessions [9]. Robotics technology can provide an overdue transformation of rehabilitation clinics from labor-intensive operations to technology-assisted operations as well as a rich stream of data that can facilitate patient diagnosis, customization of the therapy, and maintenance of patient records (at the clinic and at home) [10]. Thus, robotic devices have been developed for human ankle rehabilitation by some research groups [11-13]. Currently, there are mainly two kinds of robot-assisted ankle rehabilitation devices: those that are wearable devices mainly aiming at improving ankle performance during gait and those that are platform based devices focusing solely on improvement of ankle performance [14-16].

However, little is known about the effects of robot-assisted therapy on ankle recovery from disabilities and the intervention most effective for a specific case. The purpose of this systematic review was to provide a comprehensive investigation and examination of published evidence on the effectiveness of robot-assisted therapy used to help human ankles recover from musculoskeletal or neurologic injuries.

## Method

### Search strategy

The literature search was limited to English-language articles (i.e., journal articles, extended abstracts, and conference proceedings) published between January 1980 and June 2012 in the following electronic databases recommended by a librarian in Auckland University: PubMed, EMBASE (Excerpta Medical database), MEDLINE (OvidSP), CDSR (Cochrane database of systematic reviews), Web of Science, Scopus, Compendex, IEEE Xplore, ScienceDirect, Wiley Online Library, Digital Dissertations, Academic Search Premier, SpringerLink. The electronic search terms were “‘Ankle*’ AND ‘Robot*’ AND (‘rehabilitat*’ or ‘treat*’)”. A free search in Google Scholar was also conducted and valuable references listed in relevant publications were screened, which made our search as systematic and complete as possible.

The two primary reviewers (MZ, TCD) were a PHD student with expertise in mechatronics system and a senior lecturer with expertise in assistive technologies of rehabilitation, respectively. The third reviewer (SX) was a professor with expertise in medical robotics, assistive and rehabilitation robots. Two of reviewers (TCD, SX) hold doctorates in their respective fields. The initial search was conducted in thirteen electronic databases by one primary reviewer (MZ) and the total number of articles identified was 686. To start with, one primary reviewer (MZ) assessed all the titles for eligibility using the screening criteria described below. MZ independently assessed all abstracts after the first round screening. Abstracts considered as meeting the inclusion criteria by MZ were automatically included in the full review. Otherwise, they were excluded. Studies one primary reviewer (MZ) was not sure whether to include or not were discussed by two primary reviewers. Discrepancies between the two primary reviewers were resolved through participation from the third reviewer and discussion among these three reviewers. A search in Google Scholar was conducted by one primary reviewer (MZ) based on therapeutic effects of ankle rehabilitation robots and the references of the included papers were also screened by MZ for any additional studies. These studies newly selected were also reviewed.

**Figure 1:**
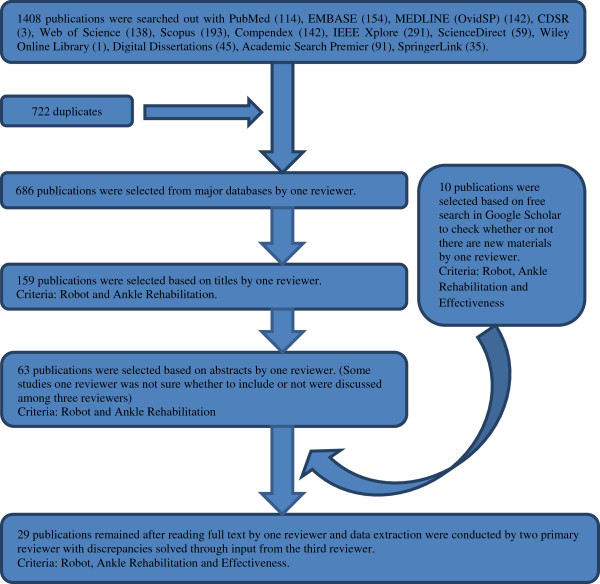
Flow diagram of selection process for final review.

Twenty-nine studies were included in the final review. Data extraction was then undertaken. The review concentrated on evidence based therapeutic effects of robot-assisted ankle rehabilitation, design types, levels of evidence and quality levels of the articles. The schematic overview of selection process with search results is shown in Figure 1.

### Inclusion and exclusion criteria

Robotics was defined as: “The application of electronic, computerized control systems to mechanical devices designed to perform human functions [17].” The American Heritage Dictionary defined a robot as a mechanical device that sometimes resembled a human and was capable of performing a variety of often complex human tasks on command or by being programmed in advance, or a machine or device that operated automatically or by remote control [18].

All trials assessing the clinical outcomes of robot-assisted ankle rehabilitation training were included. These included participants who sustained any grade of ankle disabilities caused by musculoskeletal or neurologic injuries. Both male and female participants from athletic and non-athletic populations were included to allow the generalisation of results to different populations. Papers involving platform based ankle rehabilitation robots or wearable ankle rehabilitation robots were included.

However, studies were excluded if participants underwent ankle surgeries or wore ankle prostheses. Animal based trials assessing humans and healthy subjects based trials assessing patients were also excluded. Studies focusing on the whole lower limb but not related to ankle recovery were excluded as well. Only English articles published in peer-reviewed journals or published as conference papers or abstracts were included.

### Organization of evidence

The data extraction form used for this study was the critical review form for quantitative studies developed by the Occupational Therapy Evidence-Based Practice Group at McMaster University, Hamilton, Ontario, Canada [19,20] provided guidelines for the reviewer to summarize information about study purpose, background literature, design category, sample size, outcome measures, treatment interventions, results and conclusions. Levels of evidence for the selected studies were assessed according to guidelines from AACPDM [21] and were evaluated by the two primary reviewers.

## Results

The search results are summarized in Figure 1. Sixty-three abstracts appeared to meet the inclusion criteria, and the associated full articles were obtained through downloading from electronic databases. Moreover, ten papers that appeared to meet the inclusion criteria were obtained from Google Scholar. Sixteen papers were excluded because they attempted to assess the effects of robot-assisted ankle rehabilitation devices by setting healthy subjects to participate the training [22-37]. A further two papers were excluded because they only validated the feasibility of robotic devices through simulations [38,39]. Eighteen papers focusing on the design of ankle rehabilitation robots without application were also excluded [11-13,40-54]. Four other papers involved descriptive review about robot-assisted lower extremity rehabilitation [14-16,55] and they were excluded. A conference paper [56] with its main content included in a journal article [13] was excluded; studies [57] and [58] involved the same patients and the former was excluded. Both [59] and [60] which focused on a novel approach to walking therapy were excluded. Another two studies [61,62] concerning the whole lower limb recovery but no ankle were excluded. Eventually, 29 papers that still met the inclusion criteria were selected for this systematic review [58,63-90]. These studies were carried out to evaluate the therapeutic effects of ankle rehabilitation robots through participation from related patients.

### Study characteristics

A total of 29 original papers, with data from 164 patients and 24 healthy participants used as control participants in different studies, met the inclusion criteria after the eventual discussion among three reviewers. 16 of these studies focused on evaluating the therapeutic effects of platform based ankle rehabilitation devices, as shown in Table 1. Another 13 papers discussed the therapeutic effects of wearable ankle rehabilitation robots (three studies involved anklebot, one was for in-bed ankle rehabilitation robot, six studies focused on ankle-foot orthoses (AFOs) and three papers involved a robotic-assisted locomotor training device partly focused on ankle rehabilitation), as shown in Table 2. The 164 patients spanned the ages 7 to 81 while the age of 30 patients in six papers [65,68,76,88,89,91] was not stated. The majority of participants sustained ankle disabilities mainly caused by musculoskeletal injuries and stroke. Additionally, traumatic or non-traumatic brain, spinal cord injuries, Cauda Equine syndrome and Guillain-Barre syndrome were also key factors for ankle disabilities.

**Table 1:** Reviewed studies of platform based ankle rehabilitation robot

**Study**	**Design**	**Subjects**	**Characteristics**	**Age**	**Intervention**	**Measures**	**Outcomes**	**Assumptions**
Single Subject Research Designs (SSRD)								
M. Girone, 2000 [80]	Level V, Case Study	N = 4	2 patients exhibited hypermobility secondary to chronic ankle instability and the other 2 presented with hypomobility as the sequelae of fractures	26-81	Rutgers Ankle prototype	Displacement and torque	The displacement of the uninvolved leg was comparable to normal ROM at the ankle with five degrees of dorsiflexion to 45 degrees of plantarflexion and that of the involved limb reflects a loss of ROM of −10 degrees of dorsiflexion and 28 degrees of plantarflexion; The maximum torque generated by the uninvolved limb was much larger (4 ft · lbs. for dorsiflexion and 8 ft · lbs. for plantarflexion) than that generated by the involved limb (0.5 ft · lbs. for dorsiflexion and 4 ft · lbs. for plantarflexion)	Increase in ROM and ankle torque can result in improvements in ankle performance and gait
J. E. Deutsch, 2001 [83]	Level IV, Single Case Series	N = 3	Musculoskeletal ankle injuries	14-56	Rutgers ankle system with a 3-D piloting of an airplane	ROM, torque generation capacity and ankle mechanical work	Task accuracy improved to 100% for Case 1; a fivefold increase in ankle power output for Case 2 and a three-fold increase for Case 3; both Case 2 and Case 3 reached 100% task accuracy	Improved task accuracy means improved ankle performance and gait
J. E. Deutsch, 2001 [82]	Level IV, Before-After, Single Case	N = 1	A left cerebral vascular accident	69	Rutgers ankle system with a 3-D piloting of an airplane	Ankle and foot mobility, force generation, coordination and the ability to walk and climb stairs	Strength, endurance,task accuracy, coordination, walking and stair-climbing ability improved over six rehabilitation sessions	Laboratory functional improvements correlate with activities of daily life
R. F. Boian, 2002 [90]	Level IV, Single Case Series	N = 3	3 patients with post-stroke	Mean age: 52	The Rutgers Ankle with two video games	Power and walking endurance	Increase in power generation for all motions and walking endurance increase for one patient	Increase in power generation and walking endurance means improved ankle performance and gait
R. F. Boian, 2003 [76]	Level IV, Single Case Series	N = 3	2 patients had normal sensation and the third had a decrease with 8/12 on the FM lower extremity sensory score	Not stated	The second version of VR-based ankle rehabilitation system	Muscle strength	Subject 1 increased strength in all four muscle groups, subject 2 in two muscle groups and subject 3 in three muscle groups	Increase in ankle muscle strength means improved ankle performance
J. E. Deutsch, 2004 [77]	Level IV, Single Case Series	N = 6	Post-stroke	41-81	A robotic device (the Rutgers Ankle was the input to the virtual environment)	Gait and elevation speed	Gait speed increased 11% (p = .08) and elevation time decreased 14% (p = .05); gait endurance increased 11%; gait and elevation speed improved from 0 to 44% and 3 to 33% respectively	Improved elevation speed means improvements in ankle performance and gait
R. W. Selles, 2005 [75]	Level IV, Single Case Series	N = 10	spasticity and/or contracture after stroke	Mean: 54.6	A feedback-controlled and programmed stretching device	ROM, muscle strength, joint stiffness, joint viscous damping, reflex excitability, walking speed, and subjective experiences	Significant improvements were found in the passive ROM, maximum voluntary contraction, ankle stiffness, and comfortable walking speed	Improved ROM, muscle strength, joint stiffness, joint viscous damping, reflex excitability, walking speed and subjective experiences means improved ankle performance and gait and all these correlate with activities of daily life
D. Cioi, 2011 [64]	Level IV, Single Case (ABA)	N = 1	A child with mild ataxic CP	7	Rutgers Ankle CP	Impairment, function and quality of life	Strength, motor control, gait function, overall function and qualify of life improved obviously	Laboratory functional improvements correlate with activities of daily life
G. C. Burdea, 2012 [84]	Level V, Case Study	N = 3	3 male children with CP	7-12	Rutgers Ankle CP	Impairment, function, quality of Life and game performance	Strength, motor control, gait function, overall function, qualify of life and game performance improved obviously	Laboratory functional improvements correlate with activities of daily life; good game performance means good ankle performance
Group Research Designs (GRD)								
L-Q. Zhang, 2002 [78]	Level IV, Before-After, Case Control	N = 9	5 healthy subjects and 4 chronic stroke patients with ankle contracture and/or spasticity	All subjects (36.8 ±12.8), 4 stroke patients (53.2 ± 7.9)	A custom-designed joint stretching device	ROM, joint stiffness, viscous damping and reflex excitability	The passive and active ROM of the ankle joint increased; joint stiffness and viscosity were reduced; reductions in reflex excitability were also observed	Increase in ROM, decreased joint stiffness, viscosity and reflex excitability will result in improvements in ankle performance and gait
J. E. Deutsch, 2007 [88]	Level IV, Before-After (Group performance)	N = 6	Post-stroke	Not stated	Rutgers Ankle prototype robot with VR	Accuracy of ankle movement, exercise duration, training efficiency, mechanical power of ankle and number of repetitions	All measures improved in the first three weeks and did not decrease during the transition	Improved ankle movement accuracy, exercise duration, training efficiency, ankle power and repetitions mean improved ankle performance and gait
K. Homma, 2007 [69]	Level IV, Case Control, Single Case	N = 5	4 healthy subjects and a male with hemiplegia	30-50	A passive exercise device for ankle dorsiflexion and plantarflexion	ROM and pressure distribution	These improvements were within the margin of the measuring error	Improved ROM means improved ankle performance
A. Mirelman, 2008 [73]	Level II, RCT	N = 18	Chronic hemiparesis after stroke	VR Group: (61.8 ± 9.94, 41–75); Robotic Group: (61 ± 8.32, 45–71)	Rutgers Ankle Rehabilitation System coupled with VR VS Rutgers Ankle Rehabilitation System alone	Velocity and distance walked	Greater changes in velocity and distance walked were demonstrated for the group trained with the robotic device coupled with the VR than training with the robot alone	Improved velocity and distance walked mean improved ankle performance and gait
P. Cordo, 2009 [67]	Level IV, Before-After	N = 11	Patients with post-stroke and severe motor disability of the lower extremity	38-75	AMES treatment device for ankles	Strength, joint position and motor function	Strength increased 10% in most ankles; joint position improved 10% in all ankles; motor function improved significantly	Improved strength, joint position and motor function will result in improvements in ankle performance and gait
Y-N. Wu, 2011 [58]	Level IV, Before-After	N = 12	Children with CP	5-15 and mean age is 8 years 6 months	A portable rehabilitation robot with computer game	PROM, AROM, dorsiflexor and plantarflexor muscle strength, selective control assessment of the lower extremity and functional outcome measures	Improvements in dorsiflexion PROM (P = .002), AROM (P = .02), and dorsiflexor muscle strength (P = .001); spasticity of the ankle musculature was reduced (P = .01); selective motor control improved (P = .005); functionally, participants improved balance (P = .0025) and increased walking distance within 6 minutes (P = .025)	Improved dorsiflexor ROM and muscle strength, decreased ankle spasticity, improved motor control improved ankle performance and gait; laboratory functional improvements in terms of balance and walking distance correlate with activities of daily life
G. Waldman, 2011 [86]	Level IV, Before-After	N = 8	Stroke survivors	50.4 ± 8.9	A portable ankle rehabilitation robot	Active dorsiflexion range, dorsiflexor muscle strength, the average MAS, STREAM and Berg Balance	Active dorsiflexion range and dorsiflexor muscle strength improved (p = 0.001 and 0.01, respectively) as well as the average MAS, STREAM, Berg Balance (p = 0.04, 0.03, 0.04)	Improved active dorsiflexion range, dorsiflexor muscle strength and the average MAS, STREAM, Berg Balance mean improved ankle performance and gait

**Table 2:** Reviewed studies of wearable ankle rehabilitation robot

**Study**	**Design**	**Subjects**	**Characteristics**	**Age**	**Intervention**	**Measures**	**Outcomes**	**Assumptions**
Single Subject Research Designs (SSRD)								
J. Furusho, 2007 [70]	Level V, Case Study	N = 1	A man (case: right ankle flaccid paralysis; height: 157 cm; weight: 44 kg)	59	An AFO with MR brake	Ankle angle, reaction force and a bending moment	In swing phase, the subject can maintain the dorsal flexion and prevent the drop foot; the subject can contact ground at heel; at contact ground, GRF doesn’t lack smoothness; maximal value of a bending moment with control is larger than one without control; walking cycle is shorter than one without control	Preventing drop foot in swing phase and slap foot at heel strike can result in gait improvement
S. Tanida, 2009 [79]	Level V, Case Study	N = 1	A patient of the Guillain-Barre syndrome (183 cm and 83.1 kg)	34	I-AFO	Ankle joint angle and reaction force	The foot clearance in the swing phase was kept effectively by preventing the drop foot and the initial contact occurred in the primary stance phase normally	Preventing drop foot effectively in swing phase means good ankle joint control and performance
Y. Ren, 2011 [68]	Level V, Case Study	N = 4	Acute post-stroke	Not stated	A wearable robot for in-bed acute stroke rehabilitation	Passive and active biomechanical properties	Changes of passive and active biomechanical properties can be detected	These changes contribute to ankle performance and gait
L. W. Forrester, 2011 [66]	Level IV, Single Case Series	N = 8	Chronic stroke	62.4 ± 10.4	A visually guided, impedance controlled, ankle robotic intervention	Ankle ROM, strength, motor control, and overground gait function	Increased target success, faster and smoother movements, walking velocity whereas durations of paretic single support increased and double support decreased	Improved target success, movement and walking velocity contribute to ankle performance and they correlate with activities of daily life
K. McGehrin, 2012 [65]	Level V, Case Study	N = 2	Sub-acute stroke	Not stated	A single session of anklebot training	Ankle motor control	Increased targeting accuracy, faster speed and smoother movements.	Improved target success, movement and walking velocity contribute to ankle performance and they correlate with activities of daily life
Group Research Designs (CRD)								
J. A. Blaya, 2004 [63]	Level IV, Before-After	N = 5	2 drop-foot subjects and 3 normal participants	62, 62, 66, 67, 67	AAFO	Occurrence of slap foot and swing phase ankle kinematics	The occurrence of slap foot was reduced and swing phase ankle kinematics more closely resembled normal compared to zero and constant control schemes	Decreased slap foot means improved ankle performance and gait
M. M. Mirbagheri, 2005 [89]	Level IV, Before-After	N = 5	Incomplete SCI	Not stated	Robotic- Assisted Locomotor Training	Reflex stiffness, ROM, peak-velocity, peak-acceleration	Reflex stiffness was significantly reduced after training; voluntary movement of ankle plantarflexion and dorsiflexion were substantially improved	Decreased ankle stiffness and increased ankle movement mean improvements in ankle performance and gait
G. S. Sawicki, 2006 [71]	Level IV, Before–After	N = 5	Chronic incomplete SCI	44.6 ± 13.4	PAFO	Push-off kinematics and muscle activation amplitude	Assistance from PAFO improved ankle push-off kinematics without large decreases in muscle activation	Improvement in push-off kinematics means improved gait function
J. Ward, 2010 [87]	Level IV, Before-After, Single Case Series	N = 3	stroke syndrome	60, 48 and 48	PAFO	Robot Assisted Gait	Six-minute walk test showed an increase in distance walked for subjects 1 and 3	Laboratory functional improvement in six-minute walk correlates with activities of daily life
L. F. Chin, 2010 [74]	Level IV, Before-After	N = 23	Both inpatients and outpatients with mobility problems secondary to an acquired brain injury	51 ± 13, 26-68	A robotic-assisted locomotor training device	Functional independence measure (FIM), the Rivermead Motor Assessment (RMA) gross function subscale and Motricity Index (MI)	FIM transfer improved (p is less than 0.05); FIM ambulation improved (p is less than 0.05); RMA improved (p is less than 0.05) and MI of ankle dorsiflexion improved (p is less than 0.05)	Laboratory functional improvement correlates with activities of daily life
k. A. Shorter, 2011 [81]	Level IV, Case Control, Single Case	N = 4	3 nondisabled male volunteer subjects and 1 male volunteer subject with a diagnosis of CES	Nondisabled volunteer subjects (26 ± 4) and a patient (51)	A novel PPAFO	PPAFO System performance characteristics and functional walking	Data from nondisabled walkers demonstrated functionality and data from an impaired walker demonstrated the ability to provide functional plantar flexor assistance	Providing functional assistance contributes to ankle rehabilitation
M. M. Mirbagheri, 2011 [72]	Level IV, Before-After	N = 10	Incomplete SCI	Not stated	Robotic- Assisted Locomotor Training	Passive stiffness, reflex stiffness and maximum voluntary contraction (MVC)	Reflex stiffness and intrinsic stiffness was respectively reduced up to 65% and 60% after LOKOMAT training; MVCs were increased up to 93% in ankle extensors and 180% in ankle flexors following 4-week training	Decreased ankle stiffness and increased ankle movement mean improvements in ankle performance and gait
A. Roy, 2011 [85]	Level IV, Before-After Case Control	N = 14	7 chronic stroke who had residual hemiparetic deficits and an equal number of age- and sex-matched nondisabled control subjects	Stroke subjects: 63.7 ± 10.5, 43–75; nondisabled subjects: 56.5 ± 7.5, 50-64	A single session of Impedance-controlled ankle robot (anklebot)	Ankle motor control	Increased targeting accuracy (21.6 ± 8.0 to 31.4 ± 4.8, p = 0.05), higher angular speeds (mean: 4.7 ± 1.5 degrees/s to 6.5 ± 2.6 degrees/s, p < 0.01, peak: 42.8 ± 9.0 degrees/s to 45.6 ± 9.4 degrees/s, p = 0.03), and smoother movements (normalized jerk: 654.1 ± 103.3 s–2to 537.6 ± 86.7 s–2, p < 0.005, number of speed peaks: 27.1 ± 5.8 to 23.7 ± 4.1, p < 0.01) while nondisabled subjects did not make significant gains except in the number of successful passages (32.3 ±7.5 to 36.5 ± 6.4, p = 0.006)	Improved target accuracy, movement and angular speed mean improvements in ankle performance and gait

Studies meeting the inclusion criteria were quantitative. Study designs included one RCT with only 18 participants, one single case design, nine before-after designs, six single case series and six case study designs [19]. The remaining six studies adopted two kinds of designs, specifically, [69,81] adopted case control design and single case design, [78,85] applied case control design and before-after design, [87] adopted a combination of single case series and before-after design, [82] applied both single case design and before-after design. [83] contained three case reports. RCTs’ essential feature is a set of clients/subjects are identified and then randomly allocated to two or more different treatment groups; for single case designs, changes in accessibility using several types of technology are compared with baseline data from the same individual using intervention sequences such as ABA, ABAB, ABACA, or ABCD; single case series designs involve more than one subject/client but evaluate pre and post treatment for that individual; before-after designs allow the evaluator to collect information about the initial status of a group of clients in terms of the outcomes of interest and then collect information again after treatment is received; in case control designs characteristic or situation of interest is compared with a control group of people who are similar in age, gender and background; case study designs involved task completion exercises without control group in order to provide descriptive information about the relationship between a particular treatment and an outcome of interest [20,21].

### Outcome measures

None of the studies involved outcomes classified using the health dimensions of the World Health Organization’s International Classification of Functioning, Disability, and Health [92]. Most studies tested outcomes in terms of either ankle performance (e.g. ankle strength, ankle range of motion (ROM) and ankle motor control) [58,64,65,68-71,76,78-80,83,85], [86,88-91] or gait functionality [63,73,74,77,81,87]. Five studies assessed both ankle performance and gait functionality to verify the effects of robot-assisted ankle rehabilitation devices [66,67,75,82,84]. One study [69] also tried to assess the device’s effectiveness through pressure distribution on the footplate, but whether pressure distribution could be used as an indicator of ankle recovery is not clear. Two studies [75,80] also considered satisfaction level of participants as the evaluation criteria.

It is difficult to know how functional improvements in laboratories correlate with activities of daily life. One study [93] showed that for people with stroke, the six-minute walk test was correlated to StepWatch monitor outputs over three days. However half of the variability in usual walking performance was not explained by clinical walking tests, and the study concluded that activity monitoring should also be included in functional assessments.

Several studies have shown that gait performance is affected by ankle muscle strength (in stroke [94] and spastic diplegia CP [95]) and ankle joint position [96]. One study [97] concluded that the isokinetic torques of the paretic ankle plantar flexors had moderate to high correlations with gait and stair-climbing speeds. Another [98] revealed that the dorsiflexors strength was the most important factor for gait velocity and dynamic spasticity was the most important determinant for gait spatial symmetry. It also showed that adequate ankle control during gait was important for normal gait pattern. To some extent, however, a functional recovery of gait can be thought of as an indicator of ankle joint functional recovery.

### Methodological quality

The level of evidence was based on the AACPDM guidelines. 28 studies were conducted with evidence no higher than level IV. Only one study was designed through RCT with only 18 participants and thus the evidence level was II [21].

### Research results

The studies were grouped into two general areas based on the type of devices used for robot-assisted ankle rehabilitation. One was platform based ankle rehabilitation robot, and the other was wearable ankle rehabilitation robot.

## Discussion

Our goal in this systematic review was to identify research describing the therapeutic effects of ankle rehabilitation robot. In total, we found 29 studies of robot-assisted ankle rehabilitation for individuals with any grade of ankle disability.

### Platform based ankle rehabilitation robots

Platform based ankle rehabilitation devices have a fixed platform and thus cannot be used during gait training [37]. Parallel mechanisms are typically used for multiple degrees of freedom (DOF) systems to reduce the size of robots. With the exception of the Stewart platform based device which is capable of six DOF motion, most researchers have opted for designs which offer two or three DOF in rotational motion.

A range of platform based devices have been developed by researchers for the purpose of ankle rehabilitation. They are usually designed to carry out various ankle rehabilitation exercises such as motion therapy and muscle strength training. Motion therapy can be divided into passive, active-assist and active exercises, each requiring a different level of participation from patients, ranging from no active effort in the passive exercises to full users driven motion in active exercises. Strength training on the other hand requires robots to apply a resistive load to impede the users’ movement to improve muscle strength.

The Rutgers Ankle Rehabilitation System was adopted in nine studies [64,73,76,77,80,82,83,88], [90]. All these studies except [73] focused on the development and effects of this ankle rehabilitation system. The study [73] used an RCT design and showed that a robot Virtual Reality (VR) system had better outcomes compared with a robot alone whose improvements were modest and did not transfer to significant functional or behavioural changes on the gait of individuals after stroke. Two studies [77,82] adopted VR Rutgers Ankle to conduct post-stroke rehabilitation and assessed the effects based on different criteria. One was gait and elevation speed and the other was ankle and foot mobility, force generation, coordination and the ability to walk and climb stairs. The results indicated that Lower Extremity (LE) rehabilitation of a post-stroke individual was promising [82] and could transfer to improve gait and elevation speed [77,90] further verified the conclusion in [82] through a two-month study in which three chronic post-stroke individuals underwent LE rehabilitation. The second version of the Rutgers Ankle robot used in [76] included VR based ankle rehabilitation with task-level haptic effects to enhance patients’ experience and alleviate boredom. Results through a single case series design with three participants indicated that strength capabilities for some ankle muscles were improved and haptic effects did not interfere with patients’ ability to use the platform. Cioi, 2011 [64] proposed an updated Rutgers Ankle CP robot to allow access by youth with CP. It was concluded that patient function and quality of life improved based on increased ankle strength and motor control after 36 rehabilitation sessions. Burdea, 2012 [84] further verified the conclusion through test on three children with CP and wider evaluation criteria. VR based telerehabilitation consisting of Rutgers Ankle and a local PC connected with a remote PC over the internet was evaluated in [88] through six post-stroke patiens. Performance in terms of accuracy of ankle movement, exercise duration and training efficiency, ankle mechanical power and number of repetitions did not decrease during the transition from the third week to the fourth week. Two studies [80,83] involved orthopaedic rehabilitation using the Rutgers Ankle haptic interface. In [80], a proof-of-concept patient trial found that this device can be used for ankle rehabilitation in patients with hyper and hypomobile ankles by comparison between the healthy ankle and the injured ankle. Furthermore, [83] presented three case reports about rehabilitation of musculoskeletal injuries using the Rutgers Ankle haptic interface. The results showed improvements in ROM, torque generation capacity and ankle mechanical work over six rehabilitation sessions. However, the evidence level of these papers involving Rutgers Ankle was relatively low.

All in all, ankle rehabilitation using VR based Rutgers Ankle as compared to the Rutgers Ankle Robot alone was encouraging based on the testing of 45 participants (a child with CP and 44 with post stroke or varying musculoskeletal ankle injuries). The main effect is likely due to the alleviation of end-users’ boredom.

Rehabilitation through ankle stretching was conducted in five studies [58,68,75,78,86]. An intelligent stretching device was developed in [78] to treat the spastic/contractured ankles of neurologically impaired patients. This device stretched the ankle safely to extreme dorsiflexion and plantarflexion position where spasticity and contracture were significant until a specified peak resistance torque was reached and then the ankle was held at the extreme position for a period of time to let stress relaxation occur before it moved to the other extreme position. This made the treatment more effective than existing methods in terms of active and passive ROM, joint stiffness, viscous damping and reflex excitability in the sample of spastic patients. Selles, 2005 [75] further verified the therapeutic effects of the intelligent stretching device through a single case series design and found improvements with more outcome measures including additional muscle strength, walking speed and subjective experience of the subjects. Waldman, 2011 [86] mentioned a portable robot used for ankle rehabilitation after stroke. Each training session in this trial included passive stretching under intelligent control and biofeedback active movement training through motivating games with the robot providing assistance or resistance as needed. After 18 training sessions, eight subjects showed improvements in active dorsiflexion range, dorsiflexion muscle strength, MAS, STREAM and Berg Balance. These improvements were still observed six weeks after the study was completed. Such a device with similar training was also used for rehabilitation of LE impairments in children with CP and results demonstrated improvements in joint biomechanical properties, motor control performance, and functional capability in balance and mobility [58]. Although numerous articles have shown significant improvements of ankle performance or gait functionality, the mode by which this is achieved is unknown. Other types of trainings have not been compared to game based robotic assistance or resistance during passive stretching under intelligent control and biofeedback active movement training. Homma, 2007 [69] developed an ankle dorsiflexion/plantarflexion exercise device with passive mechanical joint and its effects were evaluated by pressure distribution on the footplate. Whether pressure distribution can be used as an indicator of recovery was not clear and the relation with biological data should be further investigated.

A novel therapeutic approach (Assisted movement with enhanced sensation (AMES)) was proposed in [67]. The effects of AMES as a treatment for hemiplegia was assessed through strength and joint positioning tests as well as motor function. For 11 subjects with severe LE motor disability, improvements on most functional tests were sustained for six months. This strategy appeared safe and effective in chronic stroke patients. However, further studies with high level evidence should be conducted to verify its therapeutic effects.

### Wearable ankle rehabilitation robots

Wearable robots known as exoskeleton robots or as powered orthoses are being developed in contrast to platform based rehabilitation robots [99]. In this review wearable ankle rehabilitation robots mainly referred to wearable anklebot and AFOs. The AFO is a single-joint orthosis designed to assist and support movements of the ankle joint. It plays an important role during human walking. The first AFO was made in the late 1960s [14]. Some original robotic orthoses have been developed around the world and some trials with patients have been conducted for assessing their effects.

Three studies [65,66,85] proposed a visually guided, impedance controlled, seated anklebot intervention for ankle rehabilitation and corresponding trial designs were conducted for assessing its effects on the paretic ankle. This control approach allowed subjects to reach targets unassisted while automatically tracking their performance; however, if subjects failed to move their ankles to reach a target in time, the robot provided assistive ankle torques [85]. In [65], two sub-acute stroke survivors performed ankle targeting movements in plantarflexion/dorsiflexion and inversion/eversion ranges with robotic assistance-as-needed and improved their ankle motor control in terms of targeting accuracy, faster speed and smoother movements. These short-term improvements were accompanied by changes in EEG power and coherence, which was possibly useful for the development of more effective anklebot training that may translate to gains in gait function. Roy, 2011 [85] evaluated short-term ankle motor performance in chronic hemiparetic stroke through a double–arm pilot study with 560 movement repetitions training only in plantarflexion/dorsiflexion ranges. Statistically significant gains were achieved as indexed by increased targeting accuracy, higher angular speed, smoother movements and number of speed peaks. Forrester, 2011 [66] adopted a similar training protocol as [85] but with a different trial design and the purpose focused on effects on hemiparetic gait after a stroke. Results showed promise for the use of a modular impedance controlled anklebot in the treatment of post-stroke hemiparesis, and seated anklebot training could reduce ankle impairment and improve gait function. However, whether seated anklebot training with a task based video game and impedance controller has better therapeutic effects compared with other ankle rehabilitation robots has not been shown.

Ren, 2011 [68] proposed a wearable robot for in-bed acute stroke rehabilitation and this device also applied passive stretching and active movement training through playing a game. Four patients participating in this trial were satisfied with this device and the positive changes of active and passive biomechanical properties were detected.

An active ankle-foot orthosis (AAFO) was developed in [63] where the impedance of the orthotic joint was modulated throughout the walking cycle to treat drop-foot gait. A before-after trial design with two drop-foot participants and three control subjects showed that a variable-impedance orthosis might have certain clinical benefits for the treatment of drop-foot gait compared to conventional AFO having zero or constant stiffness joint behaviours. University of Michigan Powered Ankle-foot orthoses’ (PAFOs’) effects were assessed through five patients with chronic incomplete spinal cord injury [71]. It has been shown that mechanical assistance from PAFOs improved ankle push-off kinematics without substantially reducing muscle activation during walking. Robotic plantarflexion assistance could be used during gait rehabilitation without promoting patients passivity. Two studies [70,79] assessed an intelligent ankle-foot orthosis (IAFO). In [70], an experiment carried out using the IAFO with developed Magneto-rheological (MR) brake and control algorithm demonstrated that drop foot in swing phase and slap foot at heel strike was prevented in control participants, which was further confirmed by a patient with Guillain-Barre Syndrome in [79]. In [87], the PAFO utilized robotic tendon technology with a single DOF resulting in ankle rotation in the sagittal plane. All participants in that study showed some positive changes in their key gait variables and these improvements were more dramatic while harnessed and using a treadmill. A portable powered ankle-foot orthosis (PPAFO) was proposed in [81] to provide untethered assistance during gait. The PPAFO provided both plantarflexor and dorsiflexor torque assistance by way of a bidirectional pneumatic rotary actuator. Healthy volunteers and a participant with a diagnosis of cauda equine syndrome participated in this research. Data from healthy walkers demonstrated functionality, and data from an impaired walker demonstrated the ability to provide functional plantarflexor assistance. Significant evidence exists to support the use of AFOs and however the therapeutic differences among them should be further investigated.

Two studies [72,89] assessed the effects of robotic-assisted locomotor training on spasticity and volitional control of the spastic ankle in persons with incomplete Spinal Cord Injury (SCI). The results showed this training was effective in reducing spasticity and improving volitional control for patients with SCI. Another study [74] collected data from both inpatients and outpatient patients with mobility problems secondary to an acquired brain injury, before and after robotic-assisted locomotor training from September 2008 to May 2009. It showed significant improvement in ankle dorsiflexion.

### Control strategies

Several categories of strategies have been reported, including, assistive, challenge-based, haptic simulation, and coaching for robotic movement training following neurologic injuries [100]. There were several studies that have examined the effects of various control strategies for robot-assisted ankle rehabilitation by unimpaired subjects. Sixteen studies [22-37] were referred for more information. All selected studies with clinical evidence showed positive therapeutic effects for patients with ankle disability. Studies like [64,76,77,82,83,90] took challenge-based robotic therapy control algorithms by providing resistance to the injured ankle during exercise and haptic simulation strategies by haptic interfaces for interacting with VR simulation. In [64], an upgrade Rutgers Ankle created a more powerful reaction force and more accurate direct kinematics, added inverse kinematics for passive training at the ankle. Three recent studies [65,66,85] proposed assistance as needed by a control mechanism applied based on the error between the target location and the proximity of the subject’s ankle, as well as the robotic torsional stiffness and damping [33]. While many studies have demonstrated that training with different robotic control strategies reduces motor impairment as assessed with various outcome measures, only a few studies have found differential benefits of particular robotic control strategies with respect to other control strategies. However, which control strategy is more effective for a certain ankle disability has not been clearly addressed yet and should be further investigated.

### Safety and reliability

Not much attention has been paid to the technologies of human robot symbiosis to date because almost all robots have been designed and constructed on the assumption that the robots are physically separated from humans [101]. In particular, safety and reliability are the underlying evaluation criteria for mechanical design, actuation, and control architectures [102]. Among all selected studies, eleven studies [63,64,68,69,72,75,76,78], [81,89] involved the safety issue when ankle rehabilitation was conducted on patients. To ensure user safety during operation, [81] used an equipment within the manufacturer’s published specifications. In [76], the interaction between the platform stiffness and the vibrations imposed safety limitations on the system. ASME device mentioned in [67] was non-invasive and the relatively small amplitudes of tendon vibration and movement make it safe to use. In [69], the given ROM was smaller than the subject’s actual ROM and the speed was set slow enough to avoid induction of spasticity for safety reasons. Appropriate procedure of emergency stop should be further examined since sudden stop may induce injury of foot. [64] proposed a snowboard foot binding on the top platform of the robot to allow safe and easy attachment to the patient’s foot. Two studies [72,89] involved locomotor training ensured subject safety by an accessible panic switch and monitored by therapists. Three other studies [68,75,78] applied velocity control for the purpose of safety. Specifically, stretching velocity slowed gradually down with increasing resistance torque or at the joint extreme positions. Zhang, 2002 [78] also mentioned the safety screws used as mechanical stop to restrict the motor ROM and a digital signal processor controller for position limit. However, few studies were conducted in terms of safety and reliability assessment. There are only two studies [75,80] whose outcome measures contained the subjective evaluation of the subjects. Subjects in [75] showed very postive subjective evaluation in terms of the comfort of stretching. Participants in [80] responded favorably to the use of the device and stated that they would enjoy having this device complementing their current rehabilitation programs.

### Optimal ankle therapy

There is no reason to believe that a “one-size-fits-all” optimal treatment exists [10]. In other words, therapy should be tailored to each patient’s needs and abilities. Robot-assisted therapy can be delivered in a variety of ways to reduce motor impairment and enhance functional motor outcomes. For instance, goal-directed therapeutic games can be designed to address motor impairments including poor coordination, impaired motor speed or accuracy, and diminished strength, as well as addressing cognitive or perceptual impairments [58,64,66,73,76,77,80,82], [83,88,90]. Depending on the participant’s impairment, robotic-assisted treatment can provide passive, active-assistive, active, and active-resistive exercises [10]. As with [10], an optimal therapy could be tailored to each stroke patient through a novel performance-based impedance control algorithm. Further investigation is needed to assess its therapeutic effects.

The question of what is the most appropriate robot-assisted ankle rehabilitation is not evident. Hogan, 2006 [103] demonstrated the form of therapy may be more important than its intensity: muscle strengthening offers no advantage over movement training. Experienced rehabilitation therapists advocated “active assist exercise” or “assistance as needed”, which refers to the principle of helping the patient perform a movement with the minimal amount of manual assistance possible [104]. Moreover, [105] showed neither ankle joint nor the subtalar joints were acting as ideal hinge joints with a fixed axis of rotation and motion of the foot-shank complex in any direction is the result of rotations at both the ankle and the subtalar joints. In detail, the contribution of the ankle joint to dorsiflexion/plantarflexion of the foot-shank complex is larger than that of the subtalar joint, the contribution of the subtalar joint to inversion/eversion is larger than that of the ankle joint, and the ankle and the subtalar joints have an approximately equal contribution to internal/external rotation movements of the foot-shank complex. Therefore, robotic movement assistance as needed given in different rotational directions should base on different joints/axes, which can be defined as “effective movement assistance as needed”.

### Limitations of this review

An attempt was made to ensure that all studies related with robot-assisted ankle rehabilitation with any grade of ankle disability were reviewed. In this review we assumed that all studies used different patients, but because some studies were conducted at the same place and at the same time, we cannot be certain whether only unrelated study populations were used. However, other research may exist in which robot or ankle was not identified as a key term within the article. For instance, some articles about ankle rehabilitation robots were probably described in terms of devices and lower extremity/lower limb. Only articles after 1980 were included in this study as robot-assisted ankle rehabilitation was very limited before then. We included published conference papers and abstracts as well as full peer-reviewed papers but did not include abstracts written in languages other than English or unpublished data. Some studies may therefore have been excluded on this basis, leading to potential incomplete search.

## Conclusion

Even though a range of robot-assisted ankle rehabilitation devices and control strategies are available for individuals with ankle disability, the most effective ankle rehabilitation device and control algorithm is still vague. This is due to a lack of universal evaluation criteria with effective outcome measures. Although using RCTs to assess effects of robots on ankle outcomes is expensive and time-consuming, so too are the robots designed to assist.

In terms of control strategies, providing too much assistance has negative consequences [100] and therefore “effective movement assistance as needed” control strategy is probably encouraging for ankle rehabilitation. Specifically, that means to assist the participant only as much as needed according to real-time ankle performance or systematically reduce its assistance as recovery progresses. This will be better if combined with VR designed to be in dynamic accordance with ankle performance. In other words, the VR system should be updated online automatically as ankle rehabilitation progresses. However, to achieve dynamic and real-time ankle rehabilitation level is extremely important for realizing “effective movement assistance as needed” control, which could be realized through dynamically evaluating ankle stiffness or based on patients’ task accuracy. The technological challenge in integrating it into VR system is significant as well.

Few studies have undergone rigorous experimental assessment with high-level evidence. Therefore, higher level trials like RCTs should be conducted aiming at assessing the therapeutic effects of robot-assisted ankle rehabilitation devices and control strategies. These trials should base on rigorous comparison with each other, and with simpler, non-robotic conventional therapy in terms of devices and control strategies. It is also necessary to set up universal evaluation criteria that should contain systematic and comprehensive outcomes to evaluate devices and control strategies, including the assessment of end-user comfort, safety and training performance. These evaluation criteria may improve the consistency of results and facilitate comparisons among ankle rehabilitation robots.

## Abbreviations

RCT: Randomized Control Trial; ACC: Accident Compensation Corporation; ROM: Range Of Motion; AACPDM: American Academy for Cerebral Palsy and Developmental Medicine; VR: Virtual Reality; LE: Lower Rehabilitation; CP: Cerebral Palsy; PROM: Passive ROM; AROM: Active ROM; AMES: Assisted Movement with Enhanced Sensation; DOF: Degree Of Freedom; PPAFO: Portable Powered AFO; AFO: Ankle-Foot Orthosis; AAFO: Active AFO; IAFO: Intelligent AFO; SCI: Spinal Cord Injury; MR: Magneto-rheological; MVC: Maximum Voluntary Contraction; FIM: Functional Independence Measure; RMA: Rivermead Motor Assessment; MI: Motricity Index; SSRD: Single Subject Research Design; GRD: Group Research Design.

## Competing interests

The authors declare that they have no competing interests.

## Authors’ contributions

MZ participated as a first reviewer in the design of the study and the data collection, data analysis and drafted the manuscript. TCD participated in the design of the review, and participated as a second reviewer in the study selection and quality assessment and helped in the data extraction and revising the manuscript. SX participated as a third reviewer to solve the discrepancies between MZ and TCD and to modify the manuscript. All authors read and approved the final manuscript.
